# Treatment Resistant Acneiform Eruption in a Young Female: A Diagnostic Pitfall

**DOI:** 10.3390/dermatopathology12030032

**Published:** 2025-09-17

**Authors:** Ioannis-Alexios Koumprentziotis, Evdoxia Panou, Antonis Tsimpidakis, Maria Gerochristou, Theodoros Iliakis, Leonidas Marinos, Alexander Stratigos, Vasiliki Nikolaou

**Affiliations:** 1 1st Department of Dermatology, “Andreas Syggros” Hospital for Skin Diseases, Medical School, National & Kapodistrian University of Athens, 16121 Athens, Greece; ev_panou@yahoo.gr (E.P.); tsimpidakis.antonis@gmail.com (A.T.); gerochristou.maria@gmail.com (M.G.); drcab@hotmail.com (T.I.); alstrat2@gmail.com (A.S.); drviknik@yahoo.com (V.N.); 2 Hematopathology Department, “Evangelismos” General Hospital, 16121 Athens, Greece; lestrand@yahoo.gr

**Keywords:** acneiform rash, cutaneous lymphoma, folliculotropic mycosis fungoides

## Abstract

A 27-year-old female with no significant medical or dermatologic history presented with a persistent acneiform eruption on the face. The patient had been treated with multiple topical and systemic anti-acne treatments with no significant improvement over a period of two years. A punch biopsy was performed on the right cheek lesion showing dense lymphocytic infiltrates of the reticular dermis with peri- and intra-follicular distribution.

## 1. Case Presentation

**Figure 1:**
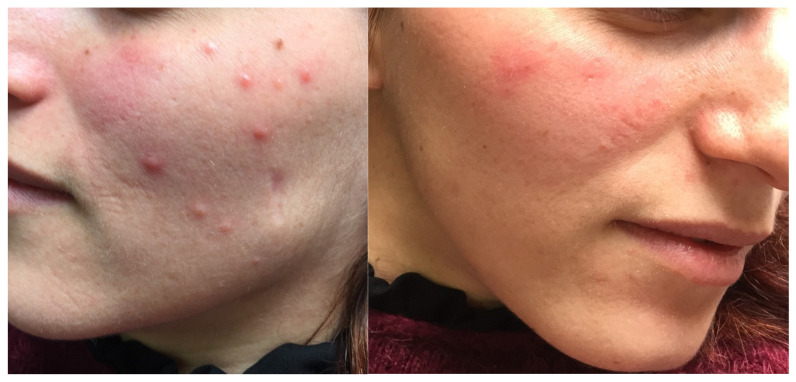
Erythematous follicular papules predominantly affecting the cheeks and chin, associated with mild pruritus.

**Figure 2:**
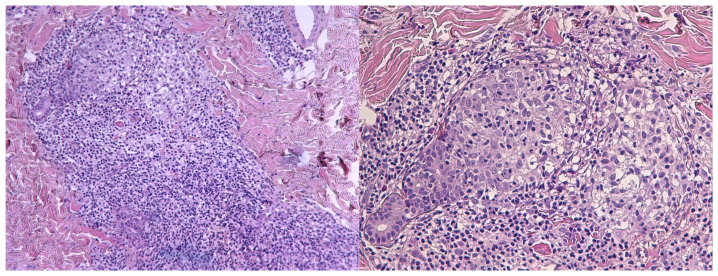
Dense lymphocytic infiltrates of atypical cells within the reticular dermis with peri- and intra-follicular distribution (H&E, 100× and 200×).

A 27-year-old female with no significant medical or dermatologic history presented with a persistent acneiform eruption on the face. Lesions first appeared at the age of 25 and were characterized by erythematous follicular papules predominantly affecting the cheeks and chin, associated with mild pruritus (Figure 1). The rash followed a relapsing–remitting course, with spontaneous erythema and episodic flares occurring throughout the year, without identifiable triggering factors. The lesions were firm on palpation, non-tender, and did not exhibit evidence of pustulation or secondary infection. No other mucocutaneous involvement was observed during the initial evaluation, including the scalp, oral mucosa, or genital region. No systemic symptoms, such as fever or weight loss, were reported. Overall, the patient was in excellent physical condition with no other systemic abnormalities except for slightly bilateral enlarged cervical lymph nodes at clinical examination. From the onset of the facial rash, the patient had been treated with multiple anti-acne topical agents such as metronidazole, clindamycin and benzoyl peroxide, azelaic acid and methylprednisolone along with systemic agents including doxycycline (200 mg/day for 4 months) and minocycline (200 mg/day for 3 months). No resolution or notable improvement was observed with any of the treatments utilized with the patient reporting a negative impact on her quality of life due to the chronicity of the lesions. Therefore, an incisional biopsy was performed to aid in the diagnosis. Histopathologic examination showed dense lymphocytic infiltrates of the reticular dermis with peri- and intra- follicular distribution (Figure 2). The infiltrating lymphocytes were small and hyperchromatic, with a predominantly perifollicular distribution.

## 2. What Is the Diagnosis?

A. Acne vulgaris
B. Papulopustular rosacea
C. Folliculotropic mycosis fungoides
D. Lupus miliaris disseminatus faciei
E. Cutaneous Sarcoidosis

## 3. Diagnosis

Folliculotropic mycosis fungoides (FMF).

## 4. Discussion

We present a case of FMF manifesting as an atypical acneiform eruption in a young adult, highlighting the diagnostic challenges of this uncommon clinical variant. The diagnosis of FMF was confirmed through two consecutive skin biopsies, both evaluated by experienced hemopathologists, along with PCR analysis revealing an identical T-cell clone in both specimens at the time of diagnosis. Further immunohistochemical studies were performed demonstrating a phenotype of CD2+, CD3+, CD4+, CD8-, CD7-, CD30- and CD20- cells. The thorough staging work-up, including blood tests and CT imaging, revealed no systemic involvement of the disease. The final patient staging was: T1bN0M0–Stage IA.

FMF is a rare distinct clinicopathologic variant of mycosis fungoides (MF), characterized by infiltration of atypical CD4+ T cells with folliculotropism, often with minimal or absent epidermotropism. Immunohistochemistry and T-cell receptor (TCR) gene rearrangement studies can aid in confirming clonality and distinguishing FMF from other benign inflammatory skin conditions [1,2,3].

Clinically, FMF frequently presents with acneiform lesions, such as follicular papules and cysts, and alopecia, with a predilection for the head and neck region. Diagnostic challenges commonly arise because FMF lesions are frequently refractory to standard acne therapies, including topical and systemic antibiotics and retinoids [1,4,5,6]. The presence of grouped follicular papules, cystic lesions, and comedones is particularly notable in FMF and can be mistaken for severe or atypical acne, especially when occurring in adults or in an unusual distribution. Moreover, especially for facial dermatoses, as in our patient’s case, a diagnostic biopsy may be withheld initially to avoid scarring and other complications when benign diseases are suspected but may be recommended in treatment refractory cases. In similar cases where lesions predominantly affect the face, the available—though limited—literature indicates that dermoscopy, as a noninvasive diagnostic modality, can assist in differentiating FMF from other inflammatory dermatoses [7,8]. Nevertheless, there is no clear evidence supporting the diagnostic utility of dermoscopy in cutaneous lymphoma or other cutaneous lymphoproliferative diseases.

Both MF and FMF can be hard to diagnose since their clinical manifestations can be very heterogenous and may mimic more benign inflammatory skin diseases. Chronic erythematous papules on the facial area present a broad differential diagnosis, with histopathological examination serving as a key tool for accurate assessment (Table 1). Although uncommon, histopathologic overlap of folliculocentric or folliculotropic lymphoid infiltrates can occur in various dermatoses, including acne vulgaris, hidradenitis suppurativa, discoid lupus erythematosus, rosacea and drug reactions among others. To confirm clonality, TCR gene rearrangement studies support the diagnosis of malignant cutaneous disorders such as FMF.

The existing literature has demonstrated a significant diagnostic delay in patients with MF [9]. This delay is a well-recognized obstacle to optimal patient management and may have a negative impact on quality of life and, in some cases, prognosis, particularly in advanced-stage disease. In a considerable number of cases, the initial diagnostic biopsy of suspicious lesions may be nonspecific and non-diagnostic warranting for repetitive biopsies. Sampling error and unnecessary postponement of subsequent biopsies are factors that seem to influence diagnostic delay and therefore multiple biopsies should be considered in patients with skin lesions highly suggestive of MF or FMF [10]. Similar cases in the literature have been reported, demonstrating patients with acneiform eruptions that have been originally misdiagnosed and have failed to respond to standard therapies including topical and oral antibiotics, topical and oral retinoids, or topical corticosteroids, before receiving the diagnosis of FMF [4,11].

FMF is classically associated with a worse clinical course and prognosis than conventional MF, particularly in patients presenting with dense dermal infiltrates, tumor-stage lesions, or advanced disease [5]. However, its clinical course is notably heterogeneous. While some patients exhibit indolent disease with patchy or flat plaque lesions, others may develop infiltrated plaques or tumors with a more aggressive clinical course. Moreover, due to the follicular localization of lesions, FMF tends to respond less favorably to standard skin-directed therapies commonly used in classic MF, such as psoralen plus UVA (PUVA). In refractory cases, alternative treatment modalities, including localized or total skin electron beam (TSEB) radiotherapy and systemic agents such as bexarotene, interferon-a and methotrexate may offer more effective disease control [1,2,3,5]. In our case, the disease followed an indolent course. The patient initially presented with facial lesions that were refractory to topical corticosteroids but ultimately achieved complete response following treatment with imiquimod 5% cream applied three times weekly for a three-month period. The decision to use imiquimod was guided by previously published reports [12,13]. However, robust evidence remains lacking, and current clinical guidelines do not recommend imiquimod for the treatment of MF.

Three years after the initial diagnosis, during her regular follow-ups, the patient developed new patchy lesions on the thighs with a mild perifollicular distribution, which were effectively managed with topical steroid monotherapy. She has remained in remission since.

This case underscores the importance of maintaining a high index of suspicion when evaluating chronic papular eruptions unresponsive to conventional therapy, particularly in younger patients without a history of severe acne. Although FMF is rare, it should be included in the differential diagnosis of adult-onset, treatment-resistant acneiform eruptions with atypical features. Careful clinical examination and the use of dermoscopy may help distinguish benign inflammatory dermatoses from cutaneous lymphoma. Early recognition—supported by timely skin biopsy, histopathologic evaluation, and immunophenotyping—is essential for accurate diagnosis. Multidisciplinary collaboration between dermatologists and experienced pathologists is critical to minimize diagnostic delays. When proper diagnosis is made, regular follow-up is also crucial, as FMF can demonstrate an unpredictable disease course with periods of remission and relapse even when diagnosed and treated at earlier stages. Finally, this case adds to the growing body of evidence suggesting that atypical and subtle presentations of cutaneous lymphomas and of other lymphoproliferative neoplasms require vigilance and high clinical suspicion to avoid long-term diagnostic delays and eventually optimize patient management.

## Figures and Tables

**Table 1:** Main clinical and histopathological findings of common diagnoses included in the differential of facial papular eruptions.

Disease	Sites Affected	Clinical Presentation	Histopathological Findings
Acne vulgaris	Face (esp. T-zone), chest, back	Comedones, papules, pustules, nodules; scarring	Dilated keratotic follicles, sebaceous gland hypertrophy, C. acnes colonization, neutrophilic inflammation; comedones, papules, pustules
Papulopustular rosacea	Central face (cheeks, nose, chin, forehead)	Central facial papules/pustules on erythematous background, flushing, telangiectasia, no comedones present	Perifollicular/perivascular lymphohistiocytic infiltrate, telangiectasia, Demodex may be present; granulomas in granulomatous subtype
Folliculotropic mycosis fungoides	Face (esp. head/neck), trunk, extremities	Follicular papules, acneiform lesions, plaques, alopecia, often pruritic, refractory to standard acne therapy	Dense perifollicular infiltrate of atypical CD4+ T-cells with folliculotropism, follicular mucinosis, possible large cell transformation
Lupus miliaris disseminatus faciei	Central face (esp. eyelids, periorbital, cheeks)	Symmetric, monomorphic, reddish-brown papules/nodules, often periorbital, scarring	Dermal epithelioid granulomas with central caseation necrosis, minimal inflammation; no mycobacterial DNA
Cutaneous Sarcoidosis	Face (esp. nose, periorbital, cheeks), other sites	Red-brown to violaceous papules/plaques, often chronic, may ulcerate or scar; may have systemic symptoms	Noncaseating granulomas in dermis, sparse lymphocytic infiltrate, no central necrosis

## Data Availability

The data supporting this material will be provided by the corresponding author upon reasonable request.

## Abbreviations

The following abbreviations are used in this manuscript:

MF	Mycosis fungoides
FMF	Folliculotropic mycosis fungoides
TCR	T-cell receptor
PUVA	psoralen plus UVA
PCR	Polymerase chain reaction
